# Telemedicine and Telehealth in Urology—What Do the ‘Patients' Think About It?

**DOI:** 10.3389/fsurg.2022.863576

**Published:** 2022-04-15

**Authors:** Nithesh Naik, B. M. Zeeshan Hameed, Sanjana Ganesh Nayak, Anshita Gera, Shreyas Raghavan Nandyal, Dasharathraj K. Shetty, Milap Shah, Sufyan Ibrahim, Aniket Naik, Nagaraj Kamath, Delaram Mahdaviamiri, Kenisha Kevin D'costa, Bhavan Prasad Rai, Piotr Chlosta, Bhaskar K. Somani

**Affiliations:** ^1^Department of Mechanical and Manufacturing Engineering, Manipal Institute of Technology, Manipal Academy of Higher Education, Manipal, India; ^2^iTRUE (International Training and Research in Uro-oncology and Endourology) Group, Manipal, India; ^3^Department of Urology, Father Muller Medical College, Mangalore, India; ^4^Department of Computer Science and Engineering, Manipal Institute of Technology, Manipal Academy of Higher Education, Manipal, India; ^5^Gandhi Medical College, Kaloji Narayana Rao University of Health Sciences, Secunderabad, India; ^6^Department of Humanities and Management, Manipal Institute of Technology, Manipal Academy of Higher Education, Manipal, India; ^7^Robotics and Urooncology, Max Hospital and Max Institute of Cancer Care, New Delhi, India; ^8^Kasturba Medical College, Manipal, Manipal Academy of Higher Education, Manipal, India; ^9^Manipal College of Pharmaceutical Sciences, Manipal, Manipal Academy of Higher Education, Manipal, India; ^10^Department of Biomedical Engineering, Manipal Institute of Technology, Manipal Academy of Higher Education, Manipal, India; ^11^Department of Urology, Freeman Hospital, Newcastle upon Tyne, United Kingdom; ^12^Department of Urology, Jagiellonian University in Krakow, Kraków, Poland; ^13^Department of Urology, University Hospital Southampton NHS Trust, Southampton, United Kingdom

**Keywords:** telemedicine, telehealth, urology, patients perspective, COVID-19

## Abstract

Telemedicine is the delivery of healthcare to patients who are not in the same location as the physician. The practice of telemedicine has a large number of advantages, including cost savings, low chances of nosocomial infection, and fewer hospital visits. Teleclinics have been reported to be successful in the post-surgery and post-cancer therapy follow-up, and in offering consulting services for urolithiasis patients. This review focuses on identifying the outcomes of the recent studies related to the usage of video consulting in urology centers for hematuria referrals and follow-up appointments for a variety of illnesses, including benign prostatic hyperplasia (BPH), kidney stone disease (KSD), and urinary tract infections (UTIs) and found that they are highly acceptable and satisfied. Certain medical disorders can cause embarrassment, social exclusion, and also poor self-esteem, all of which can negatively impair health-related quality-of-life. Telemedicine has proven beneficial in such patients and is a reliable, cost-effective patient-care tool, and it has been successfully implemented in various healthcare settings and specialties.

## Introduction

**Figure 1:**
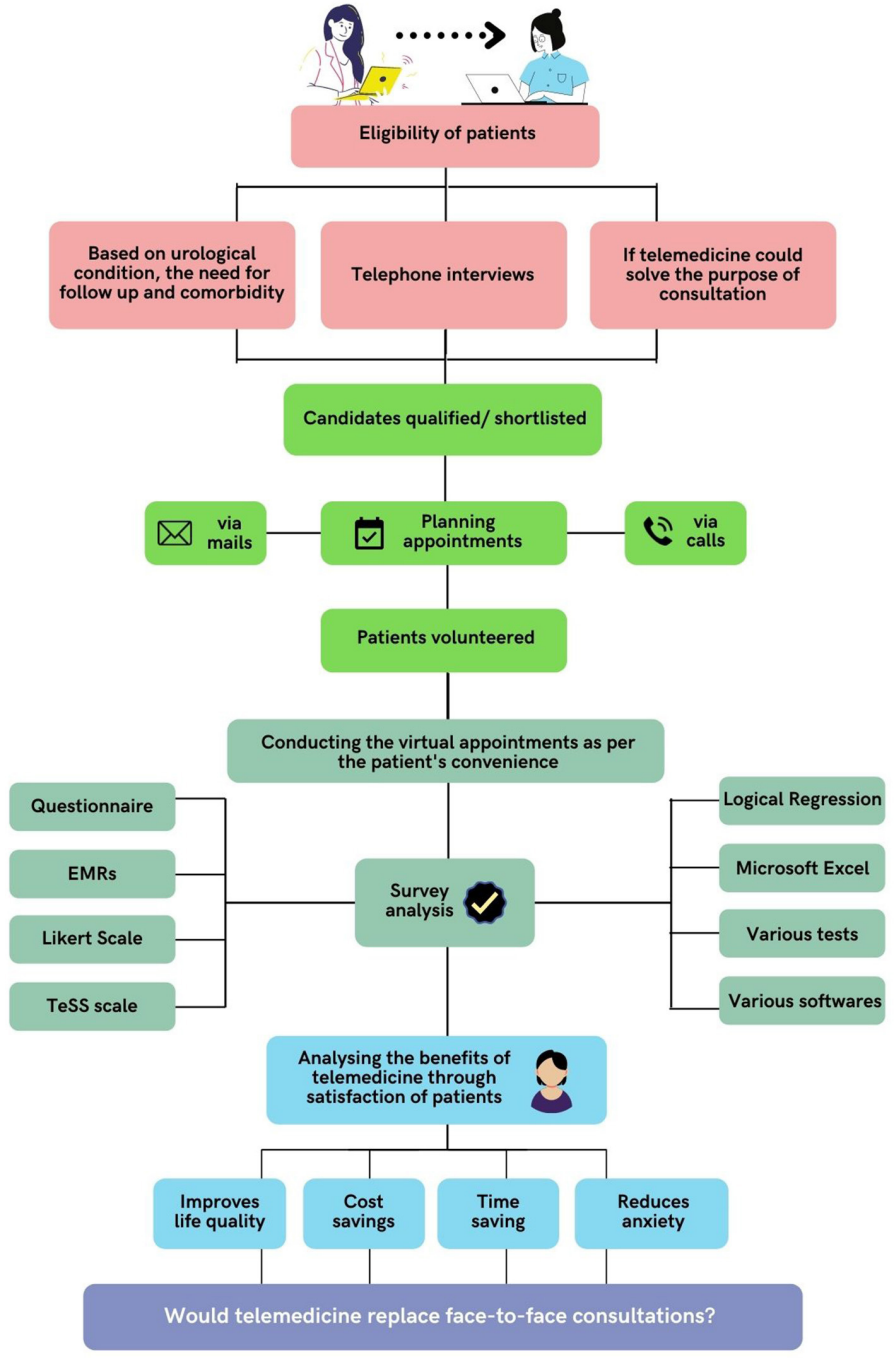
Process flow of telehealth/telemedicine tools for patient consultation.

Telehealth is a rapidly developing healthcare that entails electronic communication between patients and clinicians, and telemedicine is a subset of telehealth (1). The WHO defines telemedicine as “the delivery of healthcare services, by all healthcare workers using the information and communication technologies for the exchange of dependable data for the diagnosis, treatment, and prevention of disease and injuries, research and evaluation, and continuing education of healthcare providers, where distance is an important aspect” (2). The telephone utilization to minimize office visits was first documented in the Lancet in 1879. Telemedicine has evolved into a wide range of forms and uses electronic devices to improve healthcare delivery in various contexts (3). Video visits (VVs) have provided a real-time audio–visual substitute to traditional in-person appointments. Such an interactive communication model has allowed clinicians, patients, and families to communicate in real-time (4). Teleclinics have been reported to be successful in post-surgery and post-cancer therapy follow-up and provide consultation services for diagnosing patients with urolithiasis (5, 6). Recent research has looked into the usage of video consulting in urology centers for hematuria referrals and follow-up appointments for a variety of illnesses, including benign prostatic hyperplasia (BPH), kidney stone disease (KSD), and urinary tract infections (UTIs), having greater levels of acceptability as well as fulfillment (7–9). Telemedicine has shown to be dependable and successful as a patient-care technique and has been effectively deployed in various healthcare settings and specialties (10–13). Figure 1 shows the process flow of telehealth/telemedicine tools that are used in patient consultation.

### Benefits of Telemedicine in Healthcare

Telehealth has piqued the interest of clinicians and decision-makers, particularly since the expanded usage of the internet, with its potential to reduce healthcare costs and improve convenience (14). It uses advanced telecommunication technology to improve healthcare accessibility and availability (15). Telemedicine has a favorable impact on patients and offers cost-saving (4, 16). Enhancements in the patients' well-being, increased healthcare access in under-served areas, reduced travel time and expenses, shorter wait times, and fewer admissions in hospitals are a few of the benefits (17–19). Telemedicine has the potential to improve healthcare outcomes in the remote areas by lowering costs and boosting access to specialized treatments (20). It also ensures better information access (21). Other indirect advantages include increased adaptability in scheduling, increased availability of space in clinics and parking lots, reduced traffic, and reduced greenhouse gas emissions (22, 23).

Shin et al. (24), in their study, examined the patient contentedness and savings and clinical results of televisits in female pelvic medicine and reconstructive surgery at an urban educational center and identified that the patients reported a variety of savings, such as 88 (48.1%) of them saved at least 1 h in travel time and 54 (28.9%) saved more than $25 on transportation costs. The patient and family burden was reduced because 43 (23%) had a healthcare issue that made it hard to go for face-to-face appointments, 37 (19.8%) said that traveling for face-to-face appointments was grueling, and 41 (21.9%) said that doing a televisit allowed them to avoid taking time off work. More than half of all patients 94(51.4%) indicated they generally spend more than half an hour in the waiting area for face-to-face sessions (24).

### Importance of Telemedicine During COVID-19

On March 20, 2020, the WHO proclaimed novel coronavirus disease-19 (COVID-19), caused by severe acute respiratory syndrome Coronavirus 2 (SARS-CoV-2), an epidemic (25). The SARS-CoV-2 infection and the burden of urgent and challenging healthcare situations and needs took varied forms around the world (26, 27). Before the pandemic, telemedicine was infrequently used for follow-up visits rather than new appointments (28). The pandemic provided an exceptional chance to evaluate the efficacy of telemedicine for both new and follow-up patient consultations, as most of these appointment sessions were conducted over the phone (29).

As the epidemic enters a more chronic phase (30), with possible peaks in the future, it is critical to strike a balance between virtual treatment and a secure environment for in-person appointments. The approach of conducting telephone interviews with approved clinical tools such as checklists and questionnaires has the potential to be widely used to manage the COVID-19 pandemic's growing limitations successfully. It is a cost-effective and resource-efficient approach that protects both patients and healthcare providers while ensuring the care of vulnerable patients. (31). In addition, telephone interviews have the potential to provide social support to patients who are lonely, worried, and afraid of abandonment as a result of the pandemic (32).

Cancer remains the leading cause of death, and individuals are unwilling to compromise on their care. When there is a need for social separation, rapid introduction of telehealth is tolerated well, with a clear “red line” for alterations in the current interactions of patients and physicians. Human dignity in uro-oncology will be preserved by balancing future telemedicine implementation with the patients' demands for personal contacts (33). Because of the immunosuppression and numerous routine hospital visits, the patients of cancer having systemic treatment may be at increased possibility of developing a critical case of COVID-19(27). Telehealth can safeguard cancer patients and healthcare providers from infections during face-to-face encounters, given the existing necessity for social and patient–physician separation (33).

### Urology, Telemedicine, and COVID-19

Telemedicine in urology can reduce patient contact, lower infection rates among staff, allow isolated urologists to continue providing care (34). It can help with urological conditions such as monitoring kidney stones, renal cysts, and long-term lower urinary tract symptoms (LUTSs) management. Ureteric colic can also be diagnosed *via* phone appointment because its clinical setting is comparatively unharmful. Symptoms and scan results can be evaluated and relayed over the phone without a requirement for physical evaluation at a follow-up appointment (35, 36).

Despite the apparent advantages of telehealth, the long-term patient outcomes of urological telehealth are unknown (37). Some urologic oncology patients, mainly those with prostate cancer, have reported high satisfaction with telemedicine. Telemedicine visits were used as a consequent endpoint in a study of metformin in prostate cancer patients (38). Patients with prostate cancer who had undergone prostatectomy were highly satisfied with telemedicine (39). Apart from the prostate cancer population, the research on remote care in urologic oncology is still lacking (40). The COVID-19 epidemic has forced major changes in the healthcare worldwide, including urology (41). The pandemic has put a significant strain on hospital resources (42). As hospitals pose a substantial infection exposure through COVID-19 positive patients and asymptomatic exposed medical workers, deferred access for patient care has been suspended chiefly (43). In the COVID-19 scenario, urology is regarded as a non-essential clinical service by the international healthcare delivery system, even though the patient's quality-of-life is significantly impacted by urological disease (44, 45).

Stay-at-home policies restricted the clinic visits, including urological visits (46). In such cases, telemedicine was a practical solution for providing patients with follow-up care (47, 48). The COVID-19 pandemic has paved way for the technological advancements in the field of healthcare. Technology assisted consultation is proven to be beneficial for the patients (40, 49, 50). “Does the National Health Service (NHS) future lie in telephone consultations?” The results and experiences of the patients using NHS urology service's with telemedicine for various urological issues included clinic types ranging from post-radical prostatectomy to PSA surveillance, general, functional, and andrology followup. Patients with lower urinary tract symptoms, hematuria, and stones were observed in the general urology clinic. Patients were not evaluated individually for telephone clinic eligibility. As a result, phone calls were used to discuss difficult management decisions and deliver bad news. During the pandemic, they discovered high levels of overall contentment with the usage of telephone appointments for urology patients. Patients in the PSA surveillance and post-radical prostatectomy clinics, on the other hand, reported significantly higher levels of satisfaction (50).

In a study for outpatient hematuria referrals, involving the implementation of teleurology with the help of teleconsultation to gather clinical information using a standardized algorithm, patients reported higher overall satisfaction and acceptance rates as an expedited evaluation strategy (7). Teleconsultation-based services increase urologic access by enabling asynchronous web-based consults and patient co-management between primary care providers and urologists. Patients in the neuro-urology unit frequently have disabilities that make travel difficult and necessitate the appearance of caregivers during medical appointments. Furthermore, these patients' follow-up generally involves multiple visits, with all of the recognized effects regarding medication adherence, contentedness in general, personal uneasiness, and general and medical healthcare expenses.

Telemedicine could be beneficial in patients attending neuro-urology appointments (51). In a recent report, clinicians used telemedicine to ensure patient follow-up at the neuro-urology department dealing with problems such as persisting urine incontinence, vesicoureteric reflux, and repetitive urinary tract infections, which are pretty common in neuro-urologic patients. Telephone consultation was used because of a delay in implementing telephone consultation (using video equipment) and cognitive impairment of a few of the patients. In conclusion, telemedicine in the area of neuro-urology was related to a significant level of patient satisfaction (52). Table 1 summarizes the recent studies on telemedicine and telehealth in urology based on patient's perspectives.

**Table 1:** Summary of recent studies on telemedicine and telehealth in urology based on patient's perspectives.

**Author**	**Objective**	**Patient sample**	**Software/tests/data analysis**	**Outcomes**
Shin et al. (24)	To assess patient satisfaction and cost-effectiveness, as well as to compare the results of women who came in for a tele visit to a female pelvic medicine and reconstructive surgery (FPMRS) clinic at an urban educational center based on their chief complaint (CC).	• Cross-sectional research on women who had a telephone consultation with an FPMRS specialist	• Survey analysis • Telephone questionnaire, • Electronic medical records	• 64.5% of the women polled had completed the survey. • 89.8% were satisfied with the television visit.
Shiff. (29)	To assess the satisfaction of patients with telemedicine consultations as a substitute for in-person consultations at Andrology-focused academic urology practice during COVID-19 epidemic.	• Appointments over the phone • Telephone questionnaire	• Survey analysis • Likert scale	• As an alternative to in-person visits, patients were mostly satisfied with telephone consultations.
Barba et al., (31)	To see patients' satisfaction with the alternate strategy as deferred access, including non-urgent outpatient consultations, were suspended during the COVID-19 pandemic.	• To explore pelvic floor symptoms, telephone interviews were held with the help of a standardized questionnaire.	• JMP 9.0 (SAS, Cary, NC, USA)	• All patients were satisfied with the telephone interview. They thought it was an acceptable tool to replace routine hospital appointments during the COVID-19 lockdown.
Rodler et al. (33)	To ascertain patients' perspectives on the use of telehealth as a pandemic response and its long-term viability.	• Discussion on virtual multidisciplinary tumor boards by means of video conference.	• 10-item Likert scales • Wilcoxon matched-pair signed-rank test • Mann-Whitney U test • Chi-square test • Software: Prism 8 (GraphPad Software)	• Patients' perceptions of COVID-19 and cancer anxiety, perspectives on means of telehealth in response to the current epidemic, and long-term acceptance were employed.
Boehm et al. (42)	To evaluate the patients' eligibility for telemedicine and examine their perspective by accessing their readiness for telemedicine during the COVID-19 epidemic.	• Patients were eligible if telemedicine could entirely solve their primary purpose for consultation. • In all, 216 patients (54.1%) qualified for telemedicine and requested a telemedical visit.	• RStudio v0.98.953 (R Project for Statistical Computing, www.R-project.org)	• Risks for a serious course of COVID-19 are usual in urology patients (94.5%). • Many patients expressed an interest in having a telemedicine consultation (84.7%).
Efthymiadis et al., (50)	To assess the satisfaction of urologist patients using telephone appointments during the COVID-19 epidemic.	• A questionnaire was sent to all patients who received a phone appointment within 1 month. • In the first instance, patients were not offered face-to-face appointments.	• A seven-question adaptation of the Telehealth Satisfaction Scale (TeSS) • Likert scale • Microsoft Excel • R statistical environment • Univariable logistic regression.	• Urologist patients are generally satisfied with the usage of telephone consultations. • Telephone consultations are better suitable for some patients and may be used more frequently in the future.
Chesnel et al. (52)	To evaluate the effectiveness and contentment of telephone consulting in neuro-urology since Patients' appointments to the department of neuro-urology were limited owing to the COVID19 outbreak.	• Scheduled medical appointments were replaced by telephone consultations during the epidemic.	• Software: R and RStudio software • Means • Percentages • Standard deviations • Univariate analysis with *t*-test or variance analysis • Linear regression model in multivariate analysis • Weight kappa (wkappa)	• In neuro-urology, telemedicine was connected to high patient satisfaction and was specified as efficient by physicians. • However, patient's satisfaction was harmed by cognitive impairment, the humiliating aspect of teleconsultation, and an inclination toward physical appointment.
Heeno et al. (53)	To gather review from patients about their telemedicine experience.	• Patients for appointments in telemedicine were selected based on their urological condition, need for follow-up, and illness.	• Study-specific questionnaire	• Patients' age, sex, and distance from the hospital weren't related to satisfaction with telephone consultations. • 85% of urological patients were generally satisfied with telephone appointments.
Margolin et al. (62)	To find out how patients and doctors feel about using telemedicine to treat genitourinary cancer. The effectiveness of telemedicine for the treatment of patients having urologic malignancies was evaluated from both the patient and provider perspectives.	• Patients who had telemedicine sessions with urology, medical oncology, or radiation oncology for the treatment of genitourinary cancers were studied in a prospective cross-sectional study.	• 5-point Likert scale • Spearman correlation coefficient • ordinal logistic regression • Stata 16.1 (StataCorp, College Station, TX) • Software: MyChart (Epic, Verona, WI) or Doximity software (San Francisco, CA).	• Patients and physicians showed high levels of satisfaction with telemedicine consultations for management of genitourinary cancers.
Bell et al. (61)	To investigate the factors that led to non-attendance at a urology telehealth appointment at a large metropolitan safety-net hospital after COVID-19 obliged the institution to switch to telehealth.	• All telehealth appointments after March 17, 2020, and for the next 8 weeks were recognized.	• Stata SE 16.1 • Logistic regression • Mean • Standard deviation • Median • interquartile range • Frequency tables • Proportions • *T*-test • Chi-square statistics	• Non-attendance at outpatient telehealth urological encounters at an urban safety-net hospital in initial phases of COVID-19 epidemic was linked to various social factors like social support and drug usage.
Kim et al. (64)	To check if non-medical professionals performing Post-Operative Check-in Phone Calls (POPC) before 48 h of outpatient pediatric urological surgeries could improve patient/ family content and reduce emergency department consultations within 30 days of the procedure by increasing email/telephone communication.	• Over the course of 8 weeks, families of children receiving ambulatory pediatric urology surgeries were included.	• Likert scale • Fisher's exact test • Mann–Whitney U test • Software: Statistical Package for Social Sciences (version 20.0.0)	• POPC by an NMP in 48 h of surgery might not have an effect on perioperative satisfaction of families of patients who underwent same-day pediatric urological surgery. • But it can help to reduce postoperative anxiety.
Gan et al. (66)	To expand telemedicine for initial and follow-up pediatric urology patient visits efficiently while meeting the expectations of both patients and parents.	• Video Visits expanded in March 2020 when the epidemic was gaining traction. • Patients who had technical reasons were excluded.	• Electronic medical record • Standardized questionnaire • Software: NVivo software (launched March 2020) (13)	• Families expressed high levels of overall satisfaction with the video visits, believing that the visit addressed the medical needs of their children.
Warda et al. (83)	To see if a phone call prior to a urodynamic study (UDS) reduced test-related stress in comparison to normal care.	• Survey of patients of at least 18 years who had lesser urinary tract dysfunction was done.	• X2 • Fisher exact • Wilcoxon rank-sum tests	• The phone call before UDS did not reduce anxiety but it improved satisfaction with pre-UDS counseling.
Vallasciani et al. (84)	To use the digital clinic approach for new recommendations to Riyadh's pediatric urology clinic, the city's major tertiary care center.	• Retrospective review of the expenditures and timing associated with the VC practice • 15-question survey	• Survey analysis	• Cost savings can be achieved through telemedicine without compromising patient safety or negatively impacting patient management.
Finazzi et al. (85)	To find out the patients' sayings regarding telephone-based urological appointments during the COVID-19 epidemic.	• A cross-sectional telephone survey among some Italian patients who were scheduled for a urological appointment.	• A four-question patient questionnaire.	• In a very stressful circumstance, a telephone consultation was shown to give high levels of satisfaction, reassure urology patients, and improve their quality of life.
Wadensten et al. (60)	To see how effective the mobile software Tät II is in helping women control their Urgent Urinary Incontinence and Mixed Urinary Incontinence	• This randomized controlled experiment consisted of women of at least 18 years old having UUI or MUI and at least 2 leakages per week. • The women who displayed red flag signs weren't allowed to participate.	• Incontinence episode frequency • Intention-to-treat analysis • ICIQ-UI SF • ICIQ-LUTSqol Module • ICIQ-OAB Module • IC scale (Incontinence Catastrophizing) • Linear mixed-model analysis • Mann-Whitney U test • Paired *t*-test • Wilcoxon signed-rank test • Chi-square test • SPSS (version 25; IBM Corp)	• Women's urgency and mixed incontinence were both improved by using the treatment app. • This application may be a useful substitute to pharmaceutical care or other conservative management when self-management is appropriate, thereby boosting access to care.
Ong et al., (86)	To see how effective a telemedicine service for ureteric colic patients is at minimizing unwanted face-to-face consultations and reducing appointment wait times	• Patients with ureteric colic that did not have elevated symptoms such as fever, acute discomfort, or hydronephrosis were involved in the study, and face-to-face appointments to review scan data were replaced with phone sessions.	• SQUIRE (Standards for Quality Improvement Reporting Excellence) quasi-experimental, interrupted time series analysis	• The enrolled patients were mostly satisfied with the new service as it saved them money and time. • Without compromising patient safety, the ureteric colic telemedicine service successfully lowered the number of face-to-face visits and review time.

### The Perspective of Different Communities/Groups

It was observed in the studies that the patients' age, gender, ethnicity, race, and distance from the hospital did not seem to be related to contentment associated with telephone consultations (52, 53). However, every healthcare system includes groups at risk of bad news, who may now face increased challenges due to a lack of digital literacy or access. Rural communities, elderly persons, ethnic/racial minority populations, people with low-socioeconomic positions, inadequate health education, and little English proficiency are more likely to face this obstacle (54–56).

### Gender

Certain health conditions can cause feelings of disgrace, social separation, and low self-esteem, all of which can affect health-related quality-of-life (HRQoL) (57–59). Many women are affected by urgent urinary incontinence (UUI) and mixed urinary incontinence (MUI), significantly influencing their QoL. As a result, it is critical to provide effective treatment alternatives that can reach many patients, and eHealth technologies are fresh new ways to assist self-management. Wadensten et al. (60), in their study, examined the usefulness of the mobile application Tät II for self-monitoring of women having UUI and MUI and concluded that it was effective as the majority of the women who were present in the therapy group were happy with their treatment (60).

In their study, Shin et al. (24) attempted to assess the patient satisfaction and cost savings and analyzed visit outcomes using a main complaint of women for a televisit to the FPMRS clinic at an urban educational center. They did a cross-sectional review of all the women who finished a televisit to an FPMRS specialist at their institution and found out that 187 out of 290 (64.5%) women who were called completed the survey, and 168 (89.8%) of them were satisfied with the televisit. In total, 88 (48.1%) ended up saving at least 1 hour, and 54 (28.9%) saved more than $25 on transportation. In total, 99 (52.9%) televisits resulted in face-to-face follow-up, with chief complaint of prolapse (odds ratio [OR] = 4.2 (1.7–10.3); *p* = 0.002), new patient (OR = 2.2 (1.2–4.2); *p* = 0.01), and Hispanic ethnicity (OR = 3.9 (1.2–13.6); *p* =.03) as considerable determinants. Because many participants were comfortable with FPMRS televisits at their urban academic center, telemedicine could become a main treatment within the FPMRS specialty even after the epidemic is gone. As more healthcare institutions adopt telemedicine, further research needs to be conducted to determine which patients will benefit the most from it (24).

### Age

During the COVID-19 pandemic, Efthymiadis et al. surveyed patient satisfaction with telephone consultations, which was evaluated using an adaptation of the Telehealth Satisfaction Scale (TeSS). Patients who received a telephone appointment during 1 month were asked to fill out a survey. The patients' responses were compared by the clinic type, age, and gender. Patients in post-radical prostatectomy and PSA surveillance clinics responded with more 'Excellent' or 'Agree' comments. A considerably higher number of 'Agree' responses to one item was associated with older age. Gender had no bearing on the responses. The study found that urologist patients were quite satisfied with the utilization of telephone consultations. (50). However, older age was independently associated with lesser completed telemedicine visits.

### Vulnerable Groups

Remarkably, common sociodemographic characteristics such as race/ethnicity, country of birth, and primary language were not linked to the chances of attending a telehealth appointment (61). Margolin et al., in their study, found no significant differences in patient satisfaction among vulnerable groups (e.g., elderly patients, Hispanic/Latino ethnicity, non-White race patients). The negative consequences of technology hurdles may be felt disproportionately by vulnerable communities, thus aggravating existing healthcare gaps (62). The usage of video for telemedicine appointments was shown to be less common in black and Latinx ethnicity people creating further disparity (63).

When the racial factor was taken into account, more than three-quarters (77%) of encounters were attended by ethnic/racial minorities, and the attended and non-attended encounters had similar racial/ethnic compositions. When other considerations were taken into account, homelessness/unstable housing had no impact on the chances of attending a telehealth visit (*p* = 0.13) (61). Less video-based telemedicine consultations were connected with the black race, lower household salary, and Latinx ethnicity (63).

### Other Factors

Bell et al. (61), in their study, indicated that the married/partnered patients were in a higher percentage of attended encounters (88, 38.6%) than non-attended encounters (18, 19.2%, *p* = 0.001). Being single/widowed/divorced, having an active substance use problem, and having a new patient consultation were all linked to decreased attendance rates in multivariable analysis. The use of a language other than English as the patient's chosen language and having Medicaid insurance were both linked to independently lesser completed telemedicine appointments (61).

### Family Satisfaction

In a prospective study, Kim et al. (64) examined whether non-medical professionals (NMPs) performing post-operative check-in phone calls within 48 h of outpatient pediatric urological operations could improve patient/family contentment and reduce superfluous service utilization by increasing telephone/email communication and minimizing emergency department visits in 30 days of the process. This study suggested that well-defined perioperative guidelines and protocols can be the more significant factor in influencing patient and family happiness during the perioperative process. Moreover, it was concluded that the patient families undergoing same-day pediatric urology surgery might not be affected by a POPC made within 48 h of surgery. However, a 48-h post-operative consultation with a non-medical professional may help to alleviate post-operative anxiety. The POPCs provided improved educational and emotional assistance to most families in the pediatric age group receiving same-day tonsillectomies (65). Moreover, Gan et al. (66), in their study (66), identified that the families were generally satisfied with video visits (median score of 10/10), indicating that they met their child's medical requirements satisfactorily. A telehealth visit was strongly recommended by 90% of families. Only 15.6% of the families reported visual or hearing problems, and 7.6% reported internet connectivity problems, suggesting that the great majority of families had no technical concerns.

### Challenges

Telemedicine came with its own sets of difficulties, such as software flaws, a lack of video-compatible devices, a lack of high-speed internet, and individual technological fluency (67). Users and service providers alike experienced a variety of technical and connectivity challenges, including the visit's poor audio and/or video quality and problems in logging into the mobile application (68). Some patient-facing health apps are difficult to use for people with little health literacy (69). Furthermore, in their design and user interface, some digital health products expressly take into consideration digital literacy, age, health literacy, and English proficiency (70, 71).

Recently introduced procedures and workflows such as mandating enrolment in an online patient website have artificially created further hurdles to telehealth, even though vulnerable people are less inclined to utilize patient portals (72, 73). Even though these are excellent techniques to ensure uptake, many healthcare systems do not supply training, teaching, or guidance to patients on using these technologies (72, 74). Moreover, according to some facilitators, the outside technical aspects associated with the technique of teleconsultation include the patient's motivation, confidentiality, and familiarity with staff, and previous experience (52).

While a video visit is appropriate for most of the visits, the restrictions of remote diagnosis pose a difficulty for specific diagnoses. For example, the testing of an undescended testicle was inconsistent, and clinicians would prefer an in-person review in the future. The necessity for radiographic imaging is yet another issue that arises but is more easily solved. In this situation, some families received studies and mailed or had them transmitted electronically to the clinical team before the scheduled video consultation, facilitating inspection. Coordination of this procedure can be time-demanding for all the parties involved (66).

Furthermore, given the COVID-19 pandemic, the patient's satisfaction during the telephone consultation may be overrated (52). The encounter was deemed to have had significant technological challenges if the patient or physician reported on the survey that they had the visit over the phone without video or that the platform did not perform well enough to complete the visit as intended (62). Despite having access to the technology of mobiles, elderly patients, those from poorer socio-economic backgrounds, and those who do not speak English fluently are less inclined to use the health technologies (75, 76).

### Barriers to Telemedicine

Despite being shown to be beneficial and successful in healthcare settings, telemedicine has limitations and obstacles. It has yet to gain widespread acceptance in the urological community because of various restrictions including patient and physician acceptability, licensure and responsibility, costs, safety, and concerns with ethical issues. A systematic review of the global challenges to telemedicine adoption found that technology-specific concerns were the major barriers to telemedicine usage, with technically challenged employees being the most commonly identified impediment (77).

The pandemic period was difficult for healthcare delivery and resulted in legislative reforms that acted as a spur for us to better comprehend this previously untapped resource. Despite regulatory and legislative improvements aimed at encouraging the use of telemedicine, the financial expense of adopting it may remain a barrier for small practices (78). This implementation will continue to need careful planning, procedures and processes, and rigorous assessment to maintain the long-term viability of telemedicine and telehealth beyond the COVID-19 pandemic. Critics of telehealth use are also concerned that it will have a negative impact on the continuity of care, stating that online interactions are impersonal and unsafe since the virtual physician lacks the advantage of a comprehensive history and physical examination to help in diagnosis and treatment (79, 80).

This is particularly true in cases of chronic illnesses and malignant diseases where a thorough physical examination is of paramount importance even from the perspective of a clinician to guide the investigations and management. Ease of access, ability to use, and the design of interface being used to provide telehealth services continue to be the major technological factors limiting the use of telemedicine.

### Future Predictions of Usage of Telemedicine in the Post COVID-19 Situation

The rapid digitalization of our communities has resulted in higher levels of mobile connectivity, including in emerging economies, so a higher uptake of telemedicine is anticipated (81). Telemedicine is better suited for long-term follow-up as well as reports on chronic illnesses, as evidenced by the findings. Moreover, as telemedicine becomes more common in the specialty, validated tools for urology patients and specific patient populations will be helpful in upcoming studies. Patients in the post-radical prostatectomy and PSA surveillance clinics reported considerably higher satisfaction levels. This suggests that telemedicine will be used to some extent when the COVID-19 epidemic is over (50). Telemedicine can be used effectively in pediatric urology for various visit types, including unfamiliar, return, and post-operative consultations, with comprehensive favorable feedback from the families (66).

However, face-to-face consultations are essential in some circumstances for a safe and full clinical assessment. So, face-to-face appointments, which are superior for breaking bad news and checking acutely ill patients, cannot be replaced by telemedicine. Telemedicine should not be used to treat diseases that require a medical assessment or extensive discussions. Due to privacy concerns, people with sexual health disorders may be less eager to participate in telemedicine (8). Many patients prefer face-to-face appointments over phone consultations for personal reasons, and this should be acknowledged while keeping patient's and practitioner's safety in mind. So, as part of a patient-centered approach, the choice of telephone and remote consultations must be made available to the relevant patients (50).

Moreover, to avoid increasingly existing disparities, recognizing people at greater possibility of non-attendance because of reasons such as inadequate digital literacy, insecure housing, or substance use disorder is critical (82). As a result, healthcare organizations will need to minimize technological barriers to increase telehealth availability and acceptance among vulnerable groups. To achieve fair access to telemedicine, physicians and healthcare institutions will need to make informed, integrated, and intentional efforts (61).

## Conclusion

Telemedicine, which refers to delivering healthcare to the patients who are not at the physician's exact location, is associated with many benefits and overall patient engagement and satisfaction. It is better suited for long-term follow-up and assessment of long-standing conditions and has played a vital part in the present global COVID-19 pandemic, delivering timely patient service while simultaneously ensuring the social distancing required to avert the transmission of infectious diseases. Being a cost-effective and resource-efficient solution, it also provides additional social support to lonely and anxious patients due to the pandemic.

However, telemedicine presents its own set of potential hurdles such as software faults, a shortage of video-compatible devices, a lack of high-speed internet, and individual expertise. Besides, the rural section, elderly, ethnic/racial minority populations, and people having minor socioeconomic status, poor-health literacy, and narrow proficiency in English are more susceptible to the technological barriers. But, the choice of having telephone and remote consultations must be made accessible to appropriate patients as part of a patient-centered strategy. Healthcare organizations will need to develop strategies to eliminate technological barriers to improve telehealth accessibility and acceptance among everyone. To achieve fair access to telemedicine, physicians and healthcare institutions will need to make informed, coordinated, and intentional efforts.

## Author Contributions

NN, DS, BH, and BS contributed to the conception and design of the study. MS, SI, SN, AG, AN, and NK organized the database. SN, AG, AN, DM, KD, and SI wrote the first draft of the manuscript. NN, DM, DS, BR, SN, and BH wrote sections of the manuscript. PC, BR, and BS critically reviewed and edited the manuscript. All authors contributed to manuscript revision, read, and approved the submitted version.

## Conflict of Interest

The authors declare that the research was conducted in the absence of any commercial or financial relationships that could be construed as a potential conflict of interest.

## Publisher's Note

All claims expressed in this article are solely those of the authors and do not necessarily represent those of their affiliated organizations, or those of the publisher, the editors and the reviewers. Any product that may be evaluated in this article, or claim that may be made by its manufacturer, is not guaranteed or endorsed by the publisher.
